# Thyroid hormones and breast cancer association according to menopausal status and body mass index

**DOI:** 10.1186/s13058-018-1017-8

**Published:** 2018-08-09

**Authors:** Carolina Ortega-Olvera, Alfredo Ulloa-Aguirre, Angélica Ángeles-Llerenas, Fernando Enrique Mainero-Ratchelous, Claudia Elena González-Acevedo, Ma. de Lourdes Hernández-Blanco, Elad Ziv, Larissa Avilés-Santa, Edelmiro Pérez-Rodríguez, Gabriela Torres-Mejía

**Affiliations:** 1 Universidad Autónoma de San Luis Potosí, Facultad de Enfermería y Nutrición, Niño Artillero #130, Zona Universitaria, C.P. 78240 San Luis Potosí, S.L.P. México; 2 Red de Apoyo a la Investigación, Universidad Nacional Autónoma de México-Instituto Nacional de Ciencias Médicas y Nutrición Salvador Zubirán, calle Vasco de Quiroga No. 15, Col. Belisario Domínguez Sección XVI, Del. Tlalpan, C.P. 14080 Ciudad de México, México; 3 Centro de Investigación en Salud Poblacional, Instituto Nacional de Salud Pública, Av. Universidad No. 655, Col. Santa María Ahuacatitlán, Cuernavaca, C.P. 62100 Morelos México; 4 Hospital de Ginecología y Obstetricia No. 4 Luis Castelazo Ayala, Instituto Mexicano del Seguro Social, Avenida Río Magdalena No. 289, Col. Tizapán, San Angel, Ciudad de México, C.P. 01090 México; 5 Department of Medicine, Division of General Internal Medicine, Institute for Human Genetics, Helen Diller Family Comprehensive Cancer Center, University of California, San Francisco, 1450 3rd St, San Francisco, CA 94143 USA; 6 Department of Epidemiology and Biostatistics, Helen Diller Family Comprehensive Cancer Center, University of California, San Francisco, 1450 3rd St, San Francisco, CA 94143 USA; 7 National Heart, Lung, and Blood Institute at the National Institutes of Health, 6701 Rockledge, Room 10188, Bethesda, MD 20892 USA; 8 Hospital Universitario “Dr José Eleuterio González”. Madero y Dr. Aguirre Pequeño, Col. Mitras, C.P. 64460 Monterrey, N.L. México; 9 Instituto Nacional de Salud Pública, Centro de Investigación en Salud Poblacional, Avenida Universidad 655, Col. Santa María Ahuacatitlán, C.P. 62100 Cuernavaca, Morelos México

**Keywords:** Thyroid hormones, Triiodothyronine, Thyroxine, Obesity, Breast cancer

## Abstract

**Background:**

Thyroxine (T4) has been positively associated with tumor cell proliferation, while the effect of triiodothyronine (T3) on cell proliferation has not been well-established because it differs according to the type of cell line used. In Mexico, it has been reported that 14.5% of adult women have some type of thyroid dysfunction and abnormalities in thyroid function tests have been observed in a variety of non-thyroidal illnesses, including breast cancer (BC). These abnormalities might change with body mass index (BMI) because thyroid hormones are involved in the regulation of various metabolic pathways and probably by menopausal status because obesity has been negatively associated with BC in premenopausal women and has been positively associated with BC in postmenopausal women.

**Methods:**

To assess the association between serum thyroid hormone concentration (T4 and T3) and BC and the influence of obesity as an effect modifier of this relationship in premenopausal and postmenopausal women, we measured serum thyroid hormone and thyroid antibody levels in 682 patients with incident breast cancer (cases) and 731 controls, who participated in a population-based case-control study performed from 2004 to 2007 in three states of Mexico. We tested the association of total T4 (TT4) and total T3 (TT3) stratifying by menopausal status and body mass index (BMI), and adjusted for other health and demographic risk factors using logistic regressions models.

**Results:**

Higher serum total T4 (TT4) concentrations were associated with BC in both premenopausal (odds ratio (OR) _per standard deviation_ = 5.98, 95% CI 3.01–11.90) and postmenopausal women (OR _per standard deviation_ = 2.81, 95% CI 2.17–3.65). In premenopausal women, the effect of TT4 decreased as BMI increased while the opposite was observed in postmenopausal women. The significance of the effect modification was marginal (*p* = 0.059) in postmenopausal women and was not significant in premenopausal women (*p* = 0.22). Lower TT3 concentrations were associated with BC in both premenopausal and postmenopausal women and no effect modification was observed.

**Conclusions:**

There is a strong association between BC and serum concentrations of TT3 and TT4; this needs to be further investigated to understand why it happens and how important it is to consider these alterations in treatment.

**Electronic supplementary material:**

The online version of this article (10.1186/s13058-018-1017-8) contains supplementary material, which is available to authorized users.

## Background

The association between thyroid hormones and the risk of breast cancer (BC) has been reported in epidemiological studies [1, 2]. A positive association has been reported between thyroxine (T4) and risk of BC, which is more pronounced in overweight and obese women [1]. Negative associations have been reported between triiodothyronine (T3) and BC among premenopausal women; in contrast, positive associations have been observed among postmenopausal women [2]. It has been shown that in in vitro studies, thyroid hormones affect the growth of BC-derived cell lines [3], lung cancer [4], and glioblastoma [5]. T4 has been shown to increase cell proliferation through the αvβ3 integrin receptor found on the plasma membrane of cells [3]. In contrast, the effect of T3 on cell proliferation has not been well-established because it differs by the type of cell line used [6–8]. These effects are important, since abnormalities in thyroid function tests have been observed in a variety of non-thyroidal illnesses, without preexisting thyroid or hypothalamic-pituitary disease [9]. Furthermore, these abnormalities might change with body mass index (BMI) because thyroid hormones are involved in the regulation of various metabolic pathways (e.g., adaptive thermogenesis and glucose metabolism) that are relevant for resting energy expenditure and changes in body weight [10, 11].

Worldwide, obesity has increased to epidemic proportions in recent years. According to the World Health Organization (WHO) in 2014, 40% of women over age 18 years were obese and 15% were overweight [12]. By 2016, in Mexico, 37% of women older than 20 years were overweight, and the prevalence of obesity was 38.6% [13]. Obesity has been linked to various chronic diseases and to the development of different types of cancer, including BC [14]. One study indicated that if the BMI had remained at 1982 levels, nearly a quarter (118,000 cases) of all obesity-related cancers in 2012 could have been avoided worldwide [15].

Obesity, as measured by BMI, has been associated with BC risk, but conflicting effects have been reported in premenopausal and postmenopausal women [16–19]. In premenopausal women, BMI is associated with decreased BC risk [20–27], whereas in postmenopausal women, it has been associated with an increased BC risk [28–30]. Recently, genetically predicted BMI was inversely associated with BC risk in both, premenopausal and postmenopausal women [31]. The mechanisms behind these associations have not been fully explained [27]. Several metabolic conditions associated with body fat can influence the BC risk differently in premenopausal and postmenopausal women [27, 32].

In a cohort study conducted in Swedish women, Tosovic et al. (2012) reported a positive association between serum concentrations of free T4 (FT4) and BC prior to diagnosis, particularly in women with a BMI ≥ 25, while for free T3 (FT3), the protective effect was higher in women with BMI < 25; however, most of the associations were not statistically significant [1]. White adipose tissue actively produces various hormones and cytokines (e.g., leptin and growth factors, among others) [33], which are important in the homeostasis and regulation of thyroid hormones [34]. Several studies in euthyroid women have reported that serum free thyroxine (FT4) concentration is inversely correlated with BMI [35–37], while FT3 has been positively associated with visceral fat [38, 39] and BMI ≥ 40 [40], negatively with body fat measured using bioimpedance [41], and not correlated with BMI [36].

In the present case-control study, we examined the association between serum concentrations of thyroid hormones and BC in 2074 Mexican women who participated in the *Cáncer de Mama* (CAMA) study. We also examined obesity as an effect modifier of this relationship in premenopausal and postmenopausal women.

## Methods

### Study population

**Fig. 1:**
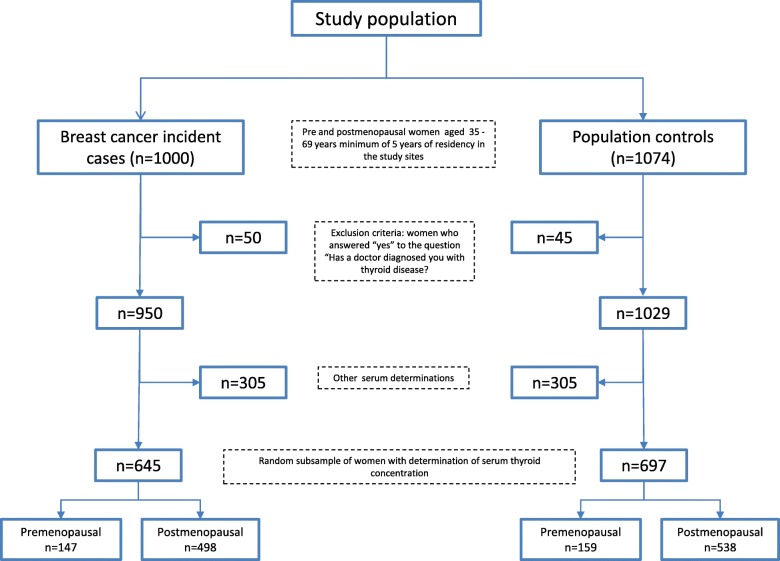
Selection from the study participants: the CAMA study, Mexico 2004–2007. A flow diagram is presented to explain the selection procedures of the participants

The present study is derived from the population-based case-control study “Risk factors for BC in Mexico: mammographic patterns, C-peptide, and growth factors, a multicenter study” (CAMA), which was conducted in three cities of Mexico (Monterrey, Veracruz, and Mexico City) from January 2004 to December 2007 [42]. In summary, the CAMA study included consecutive women with incident BC (cases (*n* = 1000)), aged 35–69 years, who were required to have had a minimum of 5 years of residency in the study cities and who were recruited from 12 public hospitals (5 from the Mexican Social Security Institute (*Instituto Mexicano de Seguro Social – IMSS*), 2 from the Institute of Security and Social Services of State Workers (*Instituto de Seguridad y Servicios Sociales de los Trabajadores de Estado – ISSSTE*), and 5 from the Ministry of Health (*Secretaría de Salud – SS*)). Nurses from the field staff were based at each hospital Monday to Friday from January 2004 to December 2007. The inclusion criteria for the cases were (a) histopathological confirmation of BC (median of 3 days between diagnosis and inclusion in the study); (b) no previous treatment (radiotherapy, chemotherapy, or antiestrogens) in the last 6 months; and (c) absence of pregnancy. The response rate of patients with BC was 94%. Controls (*n* = 1074) were selected based on a probabilistic multistage design and were randomly selected considering the catchment area of each of the participating hospitals. Mammography was performed and women with Breast Imaging Reporting and Data System (BI-RADS) categories I and II were included in the study. The response rate of controls was 87%. The control group was matched to the patients according to quinquennial age, health institution affiliation, and state of residence. In-person interviews were conducted at the hospital with the patients and at the homes of the controls to obtain information on their sociodemographics, reproductive health, breast pathology, lifestyle, and co-morbidities. Information on the perception of body image in different stages of life (six stages) was also included. The perception was measured with the use of six pictograms that represent silhouettes from very thin to very obese. In both the patients and the controls, blood samples and anthropometric measurements were obtained at participating hospitals by personnel following standardized procedures and  who were blinded to the study hypotheses. For the analyses of the present study, out of 1000 cases and 1070 controls we excluded 50 and 45 women, respectively, because they answered “yes” to the question “Has a doctor diagnosed you with thyroid disease?” In the remaining 950 cases and 1029 controls, we determined serum thyroid hormone concentrations in a random subsample of 645 cases, among whom 3 % had in situ BC, and 697 controls (Fig. 1). Characteristics (e.g., age, residence, breastfeeding, first-degree BC family history, BMI, and parity) of the subsample and the total population from the CAMA study were not statistically different (data not shown).

### Blood measurements

Blood samples were obtained from the participants after they had fasted for at least 8 h. The samples were centrifuged at 3200 rpm at room temperature for 15 min, and the serum was separated and maintained at − 20 °C for 3 weeks and then at − 80 °C until use. Total concentrations of total T3 (TT3), total T4 (TT4), thyroid stimulating hormone (TSH), thyroglobulin (Tg), and thyroid peroxidase (TPO) and thyroglobulin (Tg) antibodies (TgAb) were determined in triplicate using a chemiluminescence assay in a Beckman Coulter UniCel DxI 800 system (Brea, CA, USA) according to the manufacturer’s instructions. The coefficients of intra-assay and inter-assay variation were less than 6% for the hormones TT3, TT4, TSH, and Tg. In addition, at the Children’s Hospital Oakland Research Institute, 106 ancestry information markers were genotyped using multiplex polymerase chain reaction (PCR) and Sequenom’s unique baseline extension methodology (Sequenom Inc., San Diego, CA, USA). The details of these markers have been published elsewhere [43, 44]. The laboratory personnel who performed the measurements were blinded to the condition (case or control) of each participant.

### Anthropometric measurements

In order to obtain high-quality body measurements, trained nurses were assessed for intra-observer and inter-observer reliability until consistent and accurate anthropometric measurements were obtained. We used validated and standardized protocols and calibrated instruments according to Lohaman’s recommendations [45]. Weight was measured using a digital scale (Tanita Corporation of America, Inc., Arlington Heights, IL, USA) and recorded to the nearest 0.1 kg. Height was measured using a stadiometer (SECA, Hamburg, Germany) to the nearest millimeter. Waist circumference was measured at the level of the navel with the patient in standing position, and the hip circumference was measured at the most prominent level of the buttocks with the woman in a standing position. The BMI was calculated as the weight (in kilograms) divided by the height (in meters) squared. The waist-to-hip ratio (WHR) was calculated by dividing the waist circumference (in centimeters) by the hip circumference (in centimeters). The cutoff points used to estimate the association for BMI, waist circumference, hip circumference, and WHR were established according to the distribution of control patients into tertiles, while the cases were assigned according to the controls’ cutoff points. Tertiles were used for the anthropometric variables because Mexican women are mostly in the overweight and obesity categories. In addition, the participating women were asked to select the silhouette that best represented their body shape (using six pictograms that represent body shape from very thin to extremely obese) at different stages of their lives (before and immediately after menarche, between 18 and 20 years of age, before their first pregnancy, between 25 and 35 years, and their current body shape at the time of the interview). The correlation between BMI and silhouette has been reported as 0.67 in adult women [46], and in the study population, the correlation was *p* = 0.69 (*p* ≤ 0.001). A trajectory analysis based on a group approach was performed as proposed by Nagin [47]; this approach is based on the identification of groups with different individual trajectories in the study population over time and looks for the most homogeneous clusters. To identify the optimal model (number of groups and trajectories), we used the Bayesian information criterion (BIC) [47, 48]. To place enough subjects in each category for the statistical analysis, the body shape silhouettes were combined into categories. At childhood, at adolescence, at age 18–20 years, and at age before first pregnancy, the thin category included the women who selected silhouette 1. Silhouette 2 was included for the median category, and for obesity, the women who selected silhouettes 3, 4, 5, and 6 were included. For body silhouettes of women aged 25–35 years and for their current body silhouette, the thin category included the women who selected silhouette 1 and 2. Silhouette 3 was included for the median category, and for obesity, the women who selected silhouettes 4, 5, and 6 were included [48].

### Diet

A semi-quantitative Food Frequency Questionnaire (FFQ) adapted from Willett [49] to the Mexican population and validated in Mexico City [49, 50] was used for the present study. To measure caloric consumption, participants were asked to report frequency of consumption of a typical serving of 104 items in the past year, and responses were converted to average daily consumption. To calculate intakes, we used the nutrient database developed by the National Institute of Nutrition in Mexico [51] and, when necessary, the US Department of Agriculture food composition tables [52].

### Physical activity

To measure physical activity, a semi-structured interview to estimate an individual’s time spent performing different physical activities (sleep and light-, moderate-, and vigorous-intensity physical activity) was applied. The interview was based on the 7-day recall questionnaire proposed by Sallis et al. (1985) [53]. For the present study, weekly hours of moderate-intensity physical activity (activities that are tiresome but that do not result in breathlessness) were used. The patients were asked to report physical activity in a typical week 1 year before the appearance of signs and symptoms, to reduce the possibility of reverse causation bias, whereas the controls were asked to report physical activity for the year prior to the survey [42].

### Statistical analysis

Descriptive statistics (medians, interquartile ranges, means, SD and proportions) were calculated for both the premenopausal and postmenopausal women who were categorized as either cases or controls. We described sociodemographic, reproductive health, anthropometry, breast pathology, lifestyle, comorbidities, thyroid function parameters and clinical characteristics of the patients (cases) in terms of clinical stage (early ≤ IIA; advanced ≥ IIB) and histological grade (1, 2, or 3).

To assess the association between BC and TT3 or TT4 serum concentrations, two logistic regression models were used, one for premenopausal and one for postmenopausal women. To build each model, bivariate models were constructed for each variable of interest and potential confounders, then variables with a *p* value ≤0.20 in the bivariate models were included in each final model. In order to build the most parsimonious models that still explain the data, we left the variables with a *p* value <0.05 [54, 55]. The dependent variable was BC (yes/no), and the independent variables of interest were TT3 and TT4, which were incorporated into the models as standardized continuous variables (Z = (x-μ)/σ). For each model, odds ratios (OR) and 95% confidence intervals (95% CI) were obtained. For continuous variables such as thyroid function parameters and calorie consumption, we estimated the odds of BC for each increase in SD.

The following are the variables that were considered as potential confounders: (a) sociodemographic variables: age (years), entitlement to a health institution (IMSS, ISSSTE, and Ministry of Health), city of residence (Mexico City, Monterrey, or Veracruz), economic index (low, medium, or high), and educational level (last complete school grade); (b) reproductive health: age at menarche (years), age at menopause (years), time of exposure to endogenous hormones (age at menopause in years to age of menarche in years), parity (number of children born alive), ever use of hormonal contraception (yes/no), age at first full-term pregnancy (years), use of hormones for menopause for more than 1 month (yes/no), and breastfeeding (months); (c) anthropometric measurements: height (cm); (d) breast pathology: personal history of benign breast disease (yes/no) and family history of BC (mother, grandmother, or sisters) (yes/no); (e) lifestyles: hours of moderate-intensity physical activity per week [42, 53], alcohol consumption (consumed on average one or more alcoholic drinks a month for a year (yes/no)), tobacco consumption (smoked at least 100 cigarettes in her lifetime (yes/no), and daily calorie intake (Kcal) [49–52, 56]; (f) percentage of indigenous ancestry informative markers [44, 57]; (g) comorbidities: diabetes mellitus diagnosed by a physician (yes/no); and (h) the other thyroid function parameters: TSH, Tg, and Tg and thyroperoxidase TPO antibodies (TPO Ab) (Z = (x-μ)/σ). The percentage of ancestry informative markers was considered as a potential confounder due to its potential association with the thyroid hormone profile [57] and because it has been associated with BC [44].

The effect modification of obesity (BMI, waist circumference, hip circumference, WHR, and trajectories according to the silhouettes) was assessed for the association between TT3 or TT4 and BC in premenopausal and postmenopausal women. Multiplicative interactions were evaluated, which considered the thyroid hormones as continuous variables, the anthropometric variables in tertiles (waist circumference (tertiles), hip circumference (tertiles), BMI (tertiles), WHR (tertiles)), and the different trajectories of weight change (constantly low, constantly mid-range, moderate increase, strong increase, or constantly high). We focus our results on the potential effect modification by BMI since this variable has been associated more consistently as a protector against BC in premenopausal women and as a risk factor in postmenopausal women [23, 58–79]. The results for the rest of the anthropometric variables are presented in additional tables. Given that missing values were lower than 5%, we did not include them in the analysis of multiple models. All models were then analyzed with goodness of fit, model specification, collinearity, and influential values tests according to the procedure proposed by Hosmer and Lemeshow [55]. The analysis was performed using the STATA v13 software (StataCorp, College Station, TX, USA).

## Results

The characteristics of the study population are presented separately for premenopausal and postmenopausal women in Table 1. Compared to controls, premenopausal patients (cases), were more likely to have completed professional and postgraduate studies (data not shown). Among postmenopausal women, a higher percentage of patients completed secondary, high school, and postgraduate studies compared with the controls (data not shown). Parity, history of benign breast disease, physical activity, alcohol consumption, and history of diabetes mellitus are associated with BC according to the literature. In both premenopausal and postmenopausal women, waist circumference and hip circumference were smaller in the patients than in the controls. BMI was lower in the patients than in the controls. Table 2, shows that in both the premenopausal and postmenopausal women, the serum TT3 concentration was lower in the patients than in the controls, whereas the serum TT4 concentration was higher in the patients than in the controls. Table 3, shows that more than 40% of the study participants were diagnosed at an advanced clinical stage.

**Table 1:** Characteristics of study participants by menopausal status in the CAMA study, Mexico, 2004–2007

	Premenopausal women, *n* = 306				Postmenopausal women, *n* = 1036			
	Cases, *n* = 147		Controls, *n* = 159		Cases, *n* = 498		Controls, *n* = 538	
	Number	(%)^a^	Number	(%)^a^	Number	(%)^a^	Number	(%)^a^
*Sociodemographic*								
Age (years)^b^	44.1	38.6–47.3	44.1	40.6–47.8	58.0	52.9–64.3	57.4	52.3–62.1
Wealth index								
Low	52	(35.4)	45	(28.3)	155	(31.1)	199	(37.0)
Medium	38	(25.9)	49	(30.8)	125	(25.1)	187	(34.8)
High	57	(38.8)	65	(40.9)	218	(43.8)	152	(28.3)
*Indigenous ancestry percent*								
0–25%	3	(2.0)	4	(2.5)	21	(4.2)	10	(1.9)
26–50%	38	(25.9)	41	(25.8)	130	(26.1)	125	(23.2)
51–75%	49	(33.3)	77	(48.4)	188	(37.8)	245	(45.5)
76–100%	18	(12.2)	33	(20.8)	79	(15.9)	139	(25.8)
*Reproductive health*								
Time of exposure to endogenous hormones (years)^b^					34	30–37	34	29–37
Parity								
None	*21*	*(14.3)*	*13*	*(8.2)*	*62*	*(12.4)*	*23*	*(4.3)*
1–3	*93*	*(63.3)*	*98*	*(61.6)*	*227*	*(45.6)*	*209*	*(38.8)*
≥ 4	*32*	*(21.8)*	*47*	*(29.6)*	*205*	*(41.2)*	*306*	*(56.9)*
Use of contraception at any point at life								
No	85	(57.8)	86	(54.1)	294	(59.0)	299	(55.6)
Yes	62	(42.2)	73	(45.9)	202	(40.6)	239	(44.4)
Use of hormones for menopause for more than 1 month								
No	–	–	–	–	380	(76.3)	456	(84.8)
Yes	–	–	–	–	114	(22.9)	81	(15.1)
Breastfeeding in months^b^	9	2–25	12	4–34	12	1–42	24	6–57
*Anthropometry*								
Waist circumference (cm)^b,c^	93.3	84.8 - 101.5	97.7	92–107.0	97.0	90.3–104.8	99.5	91.65–108.0
Hip circumference (cm)^b,c^	103.1	97.2 - 111.0	107.9	102 - 115.7	106.25	100.3–114.6	108.0	101–117.0
Height (cm)^b,c^	154.5	150.5 - 158.5	153.8	149.7 - 158.0	151.8	147.3–156.0	150.5	146.7–154.4
Weight (kg)^b,c^	65.8	59.5 - 74.2	70.2	64.0–79.6	67.2	60.2–76.6	67.8	60.4–77.4
Body mass index (weight in kg/height squared)^b,c^	27.4	24.9–31.5	29.8	27.1–33.4	29.2	26.4–33.2	30.2	27.1–33.8
Waist-hip ratio^b,c^	0.90	0.86–0.94	0.91	0.87–0.95	0.91	0.87–0.96	0.91	0.87–0.97
*Silhouette trajectory*								
Group 1, constantly low	19	(12.9)	28	(17.6)	76	(15.3)	94	(17.5)
Group 2, constantly mid-range	57	(38.8)	56	(35.2)	199	(40.0)	200	(37.2)
Group 3, moderate increase	27	(18.4)	32	(20.1)	101	(20.3)	117	(21.7)
Group 4, Strong increase	41	(27.9)	37	(23.3)	104	(20.9)	113	(21.0)
Group 5, constantly high	3	(2.0)	6	(3.8)	18	(3.6)	14	(2.6)
*Breast pathology*								
Personal history of benign breast disease								
No	126	(85.7)	140	(88.1)	425	(85.3)	506	(94.1)
Yes	20	(13.6)	16	(10.1)	65	(13.1)	27	(5.0)
Family history of breast cancer (mother, grandmother, and sisters)								
No	137	(93.2)	148	(93.1)	466	(93.6)	525	(97.6)
Yes	10	(6.8)	11	(6.9)	32	(6.4)	13	(2.4)
*Lifestyle*								
Hours of moderate-intensity physical activity per week^b,d^	9.0	3.5–18.0	12.0	2.0–25.5	5.5	1.0–10.5	12.0	2.0–22.0
Consumed on average one or more alcoholic drinks a month for a year								
No	108	(73.5)	139	(87.4)	403	(80.9)	472	(87.7)
Yes	39	(26.5)	18	(11.3)	70	(14.1)	47	(8.7)
Smoked at least 100 cigarettes in her lifetime								
No	119	(81.0)	113	(71.1)	370	(74.3)	442	(82.2)
Yes	28	(19.0)	46	(28.9)	128	(25.7)	96	(17.8)
Daily total consumption of calories (Kcal)^b,d^	2262.6	1776.1 - 2704.1	1853.0	1514.8–2302.6	2014.9	1631.1–2557.1	1748.5	1396.0–2160.4
*Comorbidities*								
Diagnosed with diabetes mellitus by a physician								
No	123	(84.4)	130	(82.4)	330	(66.3)	399	(74.2)
Yes	15	(10.2)	18	(11.3)	126	(25.3)	107	(19.9)

*CAMA study* Risk factors for breast cancer in Mexico: mammographic patterns, C peptide, and growth factors, a multicenter study

^a^Percentages do not necessarily add up to 100% due to missing values

^b^Values correspond to the median and interquartile range

^c^Missing values in percentage: waist: premenopausal women: cases 0%, controls 3.1%; postmenopausal women: cases 3.8%, controls 1.9%; height: premenopausal women: cases 2%, controls 0%; postmenopausal women: cases 0%, controls 0%; body mass index: premenopausal women: cases 2%, controls 0%; postmenopausal women: cases 0%, controls 0%; waist-to-hip ratio: premenopausal women: cases 2.0%, controls 3.1%; postmenopausal women: cases 3.8%, controls 2.0%

^d^Missing values: hours of moderate-intensity physical activity per week: premenopausal women: cases 0%, controls 0%; postmenopausal women: cases 0.2%, controls 0%; daily total consumption of calories (Kcal): premenopausal women: cases 10.9%, controls 10.1%; postmenopausal women cases 5.6%, controls 7,6%

**Table 2:** Thyroid function parameters of study participants by menopausal status in the CAMA study, Mexico, 2004–2007

	Premenopausal women, *n* = 306				Postmenopausal women, *n* = 1036			
	Cases, *n* = 147		Controls, *n* = 159		Cases, *n* = 498		Controls, *n* = 538	
	Median	Interquartile range	Median	Interquartile range	Median	Interquartile range	Median	Interquartile range
*Thyroid function parameters*								
Total triiodotyronine (TT3) nmol/L^a^	1.6	1.3–1.9	2.4	1.9–2.8	1.7	1.4–2.1	2.6	2.1–3.0
Mean (SD)	1.7	0.5	2.4	0.6	1.8	0.7	2.6	0.6
Total thyroxin (TT4) nmol/L^a^	103.4	87.4–123.1	93.1	83.7–109.7	104.6	89.8–122.3	96.7	84.4–112.2
Mean (SD)	107.3	27.3	97.2	22	108.6	29.5	100.5	25.7
TSH μUI/mL^a^	1.6	1.1–2.1	1.7	1.1–2.3	1.8	1.1–2.8	1.8	1.1–2.9
Mean (SD)	1.8	1.4	2.3	3.3	2.9	9.6	2.9	6.8
Thyroglobulin ng/mL^a^	6.4	4.0–10.2	7.1	4.5–12.2	7.4	3.9–14.5	7.4	4.0–14.5
Mean (SD)	8.9	9.3	9.9	10.9	17.8	70.1	15.9	47.1
Anti-peroxidase antibodies UI/mL^b^	0.1	0.5–2.5	1.10	0.6–3.1	1.1	0.6–3.9	1.1	0.6–3.5
Mean (SD)	46.4	266.2	222.3	1373.8	111.1	444.6	95.0	346.3
Anti-thyroglobulin antibodies^b,c^								
Negative	73	(49.7)	59	(37.1)	135	(27.1)	133	(24.7)
Positive	71	(48.3)	100	(62.9)	221	(44.4)	240	(44.6)
Median UI/mL (interquartile range)^d^	0.9	0.3–3.1	1.0	0.04–3.0	1.0	0.3–4.6	0.8	0.4–2.8
Mean (SD)^d^	17.7	106.4	4.7	9.8	23.1	165.1	14.4	102.4

*CAMA study* Risk factors for breast cancer in Mexico: mammographic patterns, C peptide, and growth factors, a multicenter study, *TSH* thyroid stimulating hormone

^a^There are no missing values for TT3, TT4, TSH and thyroglobulin

^b^Results correspond to 147 cases and 159 controls (premenopausal women), respectively; and to 356 cases and 373 controls (postmenopausal)

^c^Number and (percentage) of women with negative/positive anti-thyroglobulin antibodies

^d^The values correspond to women with positive results for anti-thyroglobulin antibodies

**Table 3:** Clinical characteristics of patients with breast cancer (cases) by menopausal status in the CAMA study, Mexico, 2004–2007

	Premenopausal cases, *n* = 147		Premenopausal cases, *n* = 498	
	Number	Percentage^a^	Number	Percentage^a^
*Clinical characteristics*				
Clinical stage				
Early (≤ IIA)	43	(29.3)	175	(35.1)
Advanced (≥ IIB)	73	(49.7)	206	(41.4)
Histological grade				
1	4	(2.7)	8	(1.6)
2	29	(19.7)	108	(21.7)
3	9	(6.1)	49	(9.8)

*CAMA study* Risk factors for breast cancer in Mexico: mammographic patterns,

C peptide, and growth factors, a multicenter study

^a^Percentages do not add up to 100% due to missing values

In the premenopausal women who were stratified by tertiles of the anthropometric variables (BMI, waist and hip measurements, and WHR), the median serum TT3 concentration was lower in the patients than in the controls, whereas TT4 concentrations were higher in the patients than in the controls, and serum TSH concentrations were similar in both groups. The same relationship was observed in postmenopausal women (data not shown).

Multiple models, minimally adjusted for age, health institution, and city of residence, showed that when all the women were analyzed, the serum concentration of the TT3 hormone was negatively associated with BC (OR _per standard deviation_ = 0.16, 95% CI 0.13–0.20), and this association was maintained when the patients were stratified into the premenopausal (OR _per standard deviation_ = 0.07, 95% CI 0.04–0.12) and postmenopausal groups (OR _per standard deviation_ = 0.20, 95% CI 0.16–0.25) (data not shown). However, the protective effect was much higher in premenopausal than in postmenopausal women. The association between serum TT4 concentration and BC was positive when all the women were analyzed (OR _per standard deviation_ = 1.71, 95% CI 1.48–1.98), and this association was maintained when the patients were stratified by menopausal status; however, stronger association was seen in premenopausal women (OR _per standard deviation_ = 1.97, 95% CI 1.38–2.82) compared with postmenopausal women (OR _per standard deviation_ = 1.71, 95% CI 1.48–1.98) (data not shown). No significant associations were found with any other thyroid function parameters (TSH, Tg, Tg Ab, or TPO Ab).

Table 4 presents the multiple model stratified by menopausal status, adjusted by BMI and Table 5 presents the models stratified by both menopausal status and BMI. In Table 4, a negative association was observed between the serum TT3 concentration and BC in both premenopausal and postmenopausal women, and a positive association was observed between the serum TT4 concentration and BC. The association was stronger in premenopausal women than in postmenopausal women for both hormones. These associations were similar when each of the remaining anthropometric variables and the trajectory of the silhouettes were independently adjusted (Additional file 1: Table S1).

**Table 4:** Associations between thyroid function tests and breast cancer adjusted by BMI in the CAMA study, Mexico, 2004–2007

	Premenopausal women^a^			Postmenopausal women^b^		
	case/control	OR	95% CI	case/control	OR	95% CI
TT3^c^	128/142	0.03	0.01–0.07	382/498	0.17	0.13–0.22
TT4^c^		5.98	3.01–11.90		2.81	2.17–3.65
BMI						
Tertile 1 (BMI < 27.88)	67/47	1.00		145/166	1.00	
Tertile 2 (BMI 27.88–32.05)	35/48	0.56	0.23–1.37	106/168	0.98	0.63–1.52
Tertile 3 (BMI ≥ 32.06)	26/47	0.28	0.11–0.75	131/164	1.16	0.75–1.80

*CAMA study* Risk factors for breast cancer in Mexico: mammographic patterns, C peptide, and growth factors, a multicenter study. *TT3* total triiodothyroxine, *TT4* total thyroxine, *BMI* body mass index

^a^Logistic regression model in premenopausal women: dependent variable, breast cancer (yes/no); independent variables, TT3 (nmol/L) and TT4 (nmol/L); potential confounders, age (years), city of residence (Mexico City (reference category), Veracruz and Monterrey), health institution (IMSS: Mexican Social Security Institute (reference category); ISSSTE: Institute of Security and Social Services of State Workers; SS: Ministry of Health), daily total consumption of calories (Kcal) and BMI (tertiles). Hormone concentrations and calorie consumption were standardized to allow interpretation of the odds of breast cancer development per increment of standard deviation, Z = (x-μ)/σ

^b^Logistic model in postmenopausal women: dependent variable, breast cancer (yes/no); independent variables, TT3 (nmol/L) and TT4 (nmol/L); potential confounders: age (years), city of residence (Mexico City (reference category), Veracruz and Monterrey), health institution (IMSS: Mexican Social Security Institute (reference category); ISSSTE: Institute of Security and Social Services of State Workers; SS: Ministry of Health), thyroid stimulating hormone (continuous), parity (continuous), consumed on average one or more alcoholic drinks a month for a year (yes/no) and smoked at least 100 cigarettes in her lifetime (yes/no), indigenous ancestry (continuous) and BMI (tertiles). Hormone concentrations and calorie consumption were standardized to allow interpretation of the odds of breast cancer development per increment of standard deviation, Z = (x-μ)/σ

^c^TT3 (mean 1.7 SD 0.5); TT4 (mean 103.4 SD 27.3)

**Table 5:** Association between thyroid function tests and breast cancer modified by obesity in the CAMA study, Mexico, 2004–2007

	Premenopausal women^a^			Postmenopausal women^b^		
	Case/control	OR	95% CI	Case/control	OR	95% CI
Multiple model stratified by BMI tertiles						
Tertile 1 (BMI < 27.88)	67/47			145/166		
TT3^c^		0.02	0.003–0.09		0.18	0.11–0.28
TT4^c^		11.97	3.43–41.80		2.62	1.67–4.09
Tertile 2 (BMI 27.88–32.05)	35/48			106/168		
TT3^c^		0.04	0.0 –0.16		0.15	0.09–0.25
TT4^c^		8.34	2.03–34.24		3.03	1.83–5.02
Tertile 3 (BMI ≥ 32.06)	26/47			131/164		
TT3^c^		0.01	0.0004–0.08		0.10	0.06–0.18
TT4^c^		2.23	0.39–12.66		3.52	2.15–5.75
*p* value for interaction between TT4 and BMI tertiles			0.22			0.059
*p* value for interaction between TT3 and BMI tertiles			0.12			0.34

*CAMA study* Risk factors for breast cancer in Mexico: mammographic patterns, peptide C, and growth factors, a multicenter study, *BMI* body mass index, *TT3* total triiodothyroxine, *TT4* total thyroxine

^a^Logistic regression model in premenopausal women: dependent variable, breast cancer (yes/no); independent variables, TT3 (nmol/L) and TT4 (nmol/L); potential confounders, age (years), city of residence (Mexico City (reference category) Veracruz and Monterrey), health institution (IMSS: Mexican Social Security Institute (reference category); ISSSTE: Institute of Security and Social Services of State Workers; SS: Ministry of Health), daily total consumption of calories (Kcal). Models are presented by each tertile of BMI. Hormone concentrations and calorie consumption were standardized to allow interpretation of the odds of breast cancer development per increment of standard deviation, Z = (x-μ)/σ

^b^Logistic regression model in postmenopausal women: dependent variable, breast cancer (yes/no); independent variables, TT3 (nmol/L) and TT4 (nmol/L); potential confounders: age (years), city of residence (Mexico City (reference category) Veracruz and Monterrey), health institution (IMSS: Mexican Social Security Institute (reference category); ISSSTE: Institute of Security and Social Services of State Workers; SS: Ministry of Health), thyroid stimulating hormone (continuous), parity (continuous), consumed on average one or more alcoholic drinks a month for a year (yes/no) and smoked at least 100 cigarettes in her lifetime (yes/no) and indigenous ancestry (continuous). Models are presented by each tertile of BMI. Hormone concentrations and calorie consumption were standardized to allow interpretation of the odds of breast cancer development per increment of standard deviation, Z = (x-μ)/σ

^c^TT3 (mean 1.7 SD 0.5); TT4 (mean 103.4 SD 27.3)

When premenopausal women were stratified by BMI (Table 5), it was observed that the association between the serum concentration of TT4 and BC decreased as BMI tertiles increased, until they were no longer significant in the upper tertile (*p* of interaction = 0.22), while the protective effect of the serum TT3 concentration was maintained in the three tertiles (*p* of interaction = 0.12). Similarly, the effects of the serum concentrations of TT3 and TT4 were evaluated based on the remainder of the anthropometric variables, and waist circumference (*p* = 0.887), hip circumference (*p* = 0.291), WHR (*p* = 0.381), and the silhouettes trajectory variable (*p* = 0.52) were not statistically significant (Additional file 1: Table S2). In postmenopausal women, stratification by BMI showed that the association between the serum TT4 concentration and BC increased as the BMI tertiles increased (*p* of interaction = 0.059) (Table 5). For the other anthropometric variables, potential effect modification was observed with hip circumference (tertile 1, OR = 2.17, CI 1.34–3.50; tertile 2, OR = 3.58. CI 2.16–5.95; tertile 3, OR = 3.47, CI 2.12–5.68, *p* of interaction = 0.02), with WHR (tertile 1, OR = 2.97, CI 1.84–4.80; tertile 2, OR = 4.43. CI 2.38–8.27; tertile 3, OR = 2.46, CI 1.60–3.80, *p* of interaction = 0.02), and with the silhouettes trajectory (constantly low, OR = 3.87, CI 1.72–8.73; constantly mid-range, OR = 2.12, CI 1.44–3.13; moderately increased, OR = 2.53, CI 1.37–4.66; strong increase, OR = 4.57, CI 2.38–8.86, *p* of interaction = 0.02), but an effect modification was not observed with waist circumference (p of interaction = 0.51). The interactions between the serum TT3 concentration and the anthropometric variables were also evaluated, but these were not statistically significant for BMI (*p* = 0.34), hip circumference (*p* = 0.49), waist circumference (*p* = 0.74), WHR (*p* = 0.38), or the silhouettes trajectory (*p* = 0.36) (Additional file 1: Table S2).

## Discussion

The addition of T4 to BC-derived cell lines has been shown to increase cell proliferation [3], while in the presence of estrogen receptor (ER)-positive BC cell lines the addition of T3 inhibits cell proliferation [7]. These effects are important, as abnormalities in thyroid function tests have been observed in a variety of nonthyroidal illness, without preexisting thyroid or hypothalamic-pituitary disease, including BC [80]. These abnormalities might change by BMI because thyroid hormones are involved in the regulation of various metabolic pathways that are relevant for resting energy expenditure [10, 11]. They could also change by menopausal status because obesity has been negatively associated with BC in premenopausal women [20–27] and has been positively associated with BC in postmenopausal women [30]. It is important to assess the modifying effect of obesity in this association because of the implications for treatment in populations in where the prevalence of obesity and thyroid dysfunction is high.

We analyzed the association between thyroid hormones and BC and the modification effects of general obesity (BMI), central or intra-abdominal obesity (waist circumference, hip circumference, and waist-hip ratio), and trajectories of change in body shape. Initially, we observed that in both the premenopausal and postmenopausal women, the serum TT4 concentration was positively associated with BC, whereas the serum TT3 concentration was inversely associated with BC. These associations were stronger in the premenopausal women. When the premenopausal women were stratified by BMI tertiles, the positive association between the serum TT4 concentration and BC decreased as the BMI tertiles increased; this association was no longer statistically significant in the highest tertile, probably due to the smaller sample size. In contrast, for the postmenopausal women in the highest tertile of BMI, the strength of the association between the serum TT4 concentration and BC was increased.

A possible explanation for our findings could be related to the difference between premenopausal and postmenopausal women with respect to the possibility or risk of the development of ER-negative (ER-) BC due to BMI [81] and due to the finding that the maintenance of increased cell proliferation caused by T4 requires ER function [82]. Studies that have investigated the association between BMI and different molecular subtypes have suggested that in premenopausal women with a BMI ≥ 25, the prevalence of luminal tumors (ER positive (ER+) or progesterone receptor-positive (PR+)), human epidermal growth factor receptor 2 positive or negative (HER2+ or HER2-) and triple-negative tumors (ER-, PR-, HER2-) is higher than in those with a BMI < 25 [83]. Harris et al. (2011) consistently showed that in this same group of women, the risk of development of ER- BC was higher in those in the upper quintiles of waist and hip circumference and of WHR than in those in the lower quintile [27]. In contrast, in postmenopausal women, compared with women with a BMI < 25, the possibility of the development of luminal BC was greater in those with a BMI ≥ 25 [83]. Additionally, a pooled analysis of 35,568 women with invasive BC who participated in 34 studies showed that in those ≤ 50 years of age, the likelihood of observing ER- tumors was higher in obese women (BMI ≥ 30 kg/m^2^) than in women who were not obese (BMI < 25 kg/m^2^), and this association was statistically significant [81]. This same association was not statistically significant in women > 50 years of age [81].

Tang et al. observed that both the T4 hormone and 17β-estradiol (E2) promoted cell proliferation through the stimulation of the mitogen-activated protein kinase (MAPK) pathway by ER and demonstrated that such proliferation requires ER function to be sustained [82]. In several cell models, it has been observed that at physiological concentrations, T4 is more active than T3 at stimulating the MAPK pathway [84–87]. In premenopausal women, the decrease in the association between TT4 and BC with increasing BMI could be explained by the lower prevalence of ER+ tumors in overweight and obese women [88, 89]. In contrast, in postmenopausal women, the increase in the association between T4 and BC with increasing BMI may be explained by the finding that overweight and obese women are at a greater risk of the development of ER+ breast tumors. However, more studies need to be performed stratifying by menopausal status and BMI. Two cohort studies reported a positive association between the serum T4 concentration and BC. However, in the aforementioned studies no effect modification was assessed for BMI and menopausal status [1, 90].

In our study we observed a negative association between serum TT3 and BC in both premenopausal and postmenopausal women. On the other hand, we did not observe an effect modification of this association by BMI. In addition, our results are consistent with previously published studies. For example, in studies of ER- cell lines that were transfected with ER, T3 inhibited cell proliferation [7]. Also, Tosovic et al., reported a non-statistically significant negative association between serum T3 and BC, independently of menopausal status [1]. That group also observed a negative association when women were stratified by menopausal status and BMI; however, it was not statistically significant [1].

The findings that we report need to be interpreted within the context of certain limitations. The number of premenopausal women was not sufficient for the identification of a statistically significant effect modification by BMI or other obesity measurements; moreover, the confidence intervals after stratification were broad, particularly for other anthropometric measurements that are presented in additional tables. Given the characteristics of Mexican women, among whom more than 70% were overweight and obese, it was not possible to use the cutoff points proposed by the WHO for anthropometric measurements; hence, we stratified the controls by tertiles for each measurement. Our study personnel were trained to measure weight and height and the other anthropometric variables using a standardized approach in both cases and controls, and in the cases, the measurements were performed at the time of the diagnosis. As BMI can be modified by the presence of cancer, this study is not free of reverse causality. However, the median number of days from the diagnosis until the women entered the study and the anthropometric variables were determined was 3 days. Additionally, we measured TT4. Free T4 (FT4) is unbound and is the active component of TT4, therefore further studies should be performed using FT4. Approximately 75% of the T4 in serum is bound to thyroid-binding globulin (TBG), and a smaller fraction is bound to transthyretin or albumin; so less than 0.1% remains free or unbound [91]. Given that postmenopausal hormone therapy and the use of hormonal contraceptives at any point in life lead to increased thyroid binding globulin (TBG) binding capacity [92], we considered adjusting for these variables in our models. However, we did not include them in the final models because they did not confound our main results. We did not include the ER status because when recruiting the patients (cases), ER status was not determined in all women. Tosovic et al., (2014) found statistically significant positive associations between higher pre-diagnostic T3 concentration and negative ER status [93]. However, further analysis needs to be performed because the sample size was very small and they did not adjust or stratify by BMI.

Our findings are congruent with previously observed altered T3 and T4 measurements in diseases such as BC [9]. In those circumstances, there is dysregulation of thyrotrophic feedback control [9], in which T3 and/or T4 are at unusual levels, but the thyroid gland does not appear to be dysfunctional. Thus, the lower levels of T3 and higher levels of T4 in BC cases may be due to the effect of BC on thyroid function rather than T3 and T4 acting as risk factors for BC. If so, these findings may still be of interest to understand whether the levels of T3 and T4 could be related to tumor stage or have other implications for prognosis.

Our findings need to be replicated in other studies, including those with larger sample sizes and to investigate other possible mechanisms by which the association between T4 and BC is potentially modified by BMI in premenopausal and postmenopausal women. In particular, prospective cohort studies in which T3 and T4 are collected prior to cancer diagnosis may be helpful in understanding the causal relationship between BC and thyroid hormones. In addition, a prospective study may also help to improve understanding of the relationship among thyroid hormones, BC, and obesity.

To the best of our knowledge, this is the first study that has evaluated the effect modification by BMI of the relationship between thyroid hormones and BC, both in premenopausal and postmenopausal women. The results of the present study open a new line of research with which to evaluate the effect modification by obesity of the association between thyroid hormones and BC.

## Conclusions

There is a strong association between BC and serum concentrations of TT3 and TT4; the latter differed by BMI and menopausal status. This needs to be further investigated to understand why it happens and how important it is to consider these alterations in treatment.

## Additional file

Additional file 1: **Table S1.** Associations between thyroid function tests and BC adjusted by anthropometric variables. CAMA Study^1^, Mexico 2004–2007. The table presents the multiple model stratified by menopausal status, adjusted by hip and waist measurements, WHR, and trajectory of the silhouettes. **Table S2.** Association between thyroid function tests and BC modified by anthropometric variables. CAMA Study^1^, Mexico 2004–2007. The table presents the multiple model stratified by menopausal status, and each of anthropometric variables (hip and waist measurements, WHR, and trajectory of the silhouettes). (DOCX 30 kb)

## Abbreviations

BC: Breast cancer
bFGF: Fibroblast growth factor
BIC: Bayesian information criterion
BMI: Body mass index
CAMA: Risk factors for breast cancer in Mexico: mammographic patterns, C peptide, and growth factors, a multicenter study
E2: 17β-estradiol
ER: Estrogen receptor
FT3: Free triiodothyronine
FT4: Free thyroxine
HER2: Human epidermal growth factor receptor 2
IMSS: Instituto Mexicano de Seguro Social
ISSSTE: Instituto de Seguridad y Servicios Sociales de los Trabajadores del Estado
MAPK: Mitogen-activated protein kinase
OR: Odds ratio
PR: Progesterone receptor
RIC: Interquartile range
SS: Secretariía de Salud
T3: Triiodothyronine
T4: Thyroxine
TBG: Thyroid binding globulin
Tg: Thyroglobulin
Tg Ab: Thyroglobulin antibodies
TPO Ab: Thyroperoxidase antibodies
TSH: Thyroid stimulating hormone
TT3: Total triiodothyronine
TT4: Total thyroxine
VEGF: Vascular endothelial growth factor
WHO: World Health Organization
WHR: Waist-to-hip ratio
