# Supplementary material for: Thyroid hormones and breast cancer association according to menopausal status and body mass index
Source: Breast Cancer Res. 2018 Aug 9;20:94. doi: 10.1186/s13058-018-1017-8 (PMC6085630; doi:10.1186/s13058-018-1017-8)
Supplement: Supplementary file 1 — Table S1. Associations between thyroid function tests and BC adjusted by anthropometric variables. CAMA Study1, Mexico 2004–2007. The table presents the multiple model stratified by menopausal status, adjusted by hip and waist measurements, WHR, and trajectory of the silhouettes. Table S2. Association between thyroid function tests and BC modified by anthropometric variables. CAMA Study1, Mexico 2004–2007. The table presents the multiple model stratified by menopausal status, and each of anthropometric variables (hip and waist measurements, WHR, and trajectory of the silhouettes). (DOCX 30 kb) [file 13058_2018_1017_MOESM1_ESM.docx]

|  |  |  | **Premenopausal women^2^** | | |  | **Postmenopausal women^2^** | | |
| --- | --- | --- | --- | --- | --- | --- | --- | --- | --- |
|  |  |  | **case/control** | **OR** | **95% CI** |  | **case/control** | **OR** | **95% CI** |
| **Multiple model 1 adjusted by waist tertiles** | | | |  |  |  |  |  |  |
|  | TT3^4^ | | 129/139 | 0.03 | 0.01 - 0.07 |  | 375/492 | 0.16 | 0.12 - 0.22 |
|  | TT4^4^ | |  | 6.17 | 3.14 - 12.12 |  |  | 2.87 | 2.21 - 3.74 |
|  | Waist circumference | |  |  |  |  |  |  |  |
|  |  | Tertil 1 (<93.05 cm) | 61/46 | 1.00 |  |  | 119/153 | 1.0 |  |
|  |  | Tertil 2 (93.06-103.05 cm) | 40/51 | 0.58 | 0.24 - 1.42 |  | 145/158 | 1.17 | 0.75 - 1.82 |
|  |  | Tertil 3 (>103.06 cm) | 28/42 | 0.36 | 0.13 - 0.99 |  | 111/181 | 0.71 | 0.45 - 1.13 |
| **Multiple model 2 adjusted by hip tertiles** | | | |  |  |  |  |  |  |
|  | TT3^4^ | | 128/141 | 0.03 | 0.01 - 0.07 |  | 376/494 | 0.16 | 0.13 - 0.22 |
|  | TT4^4^ | |  | 6.71 | 3.35 - 13.44 |  |  | 2.88 | 2.21 - 3.74 |
|  | Hip circumference | |  |  |  |  |  |  |  |
|  |  | Tertil 1 (<103.00 cm) | 66/44 | 1.00 |  |  | 126/162 | 1.00 |  |
|  |  | Tertil 2 (103.01-112.00cm) | 32/51 | 0.52 | 0.21 - 1.30 |  | 132/161 | 1.42 | 0.91 - 2.23 |
|  |  | Tertil 3 (≥112.03 cm) | 30/46 | 0.24 | 0.09 - 0.66 |  | 118/171 | 1.13 | 0.71 - 1.79 |
| **Multiple model 3 adjusted by WHR tertiles** | | | |  |  |  |  |  |  |
|  | TT3^4^ | | 128/139 | 0.03 | 0.01 - 0.07 |  | 376/491 | 0.16 | 0.12 - 0.21 |
|  | TT4^4^ | |  | 5.66 | 2.93 - 10.95 |  |  | 2.89 | 2.22 - 3.76 |
|  | Waist Hip ratio | |  |  |  |  |  |  |  |
|  |  | Tertil 1 (WHR <.888) | 48/49 | 1.00 |  |  | 135/148 | 1.00 |  |
|  |  | Tertil 2 (WHR 0.888-.936) | 47/47 | 0.91 | 0.37 - 2.22 |  | 106/158 | 0.62 | 0.39 - 0.99 |
|  |  | Tertil 3 (WHR >.936) | 33/43 | 0.78 | 0.29 - 2.06 |  | 135/185 | 0.49 | 0.31 - 0.79 |
| **Multiple model 4 adjusted by silhouette trajectory** | | | |  |  |  |  |  |  |
|  | TT3^4^ | | 130/143 | 0.02 | 0.01 - 0.06 |  | 391/502 | 0.16 | 0.12 - 0.21 |
|  | TT4^4^ | |  | 6.12 | 3.09 - 12.13 |  |  | 2.81 | 2.17 - 3.63 |
|  | ***Silhouette trajectory*** | |  |  |  |  |  |  |  |
|  |  | Group 1. Constantly low | 16/26 | 1.00 |  |  | 58/88 | 1.00 |  |
|  |  | Group 2. Constantly mid-range | 50/52 | 1.93 | 0.63 - 5.94 |  | 153/185 | 1.22 | 0.72 - 2.06 |
|  |  | Group 3. Moderate increase | 25/28 | 2.45 | 0.69 - 8.74 |  | 80/112 | 1.48 | 0.82 - 2.69 |
|  |  | Group 4. Strong Increase | 36/34 | 3.1 | 0.93 - 10.33 |  | 85/104 | 0.91 | 0.50 - 1.64 |
|  |  | Group 5. Constantly High | 3/6 | 6.75 | 0.63 - 72.13 |  | 15/13 | 1.26 | 0.43 - 3.68 |

Table S1. Associations between thyroid function tests and BC adjusted by anthropometric variables. CAMA Study^1^. Mexico 2004-2007

^1^ CAMA: risk factors for breast cancer in Mexico: mammographic patterns, peptide C, and growth factors, a multicenter study

^2^ Logistic model in premenopausal women: Dependent variable: breast cancer (yes/no); Independent variables: TT3 (nmol/L) and TT4 (nmol/L); potential confounders: All models are adjusted by: age (years), city of residence (Mexico City [reference category] Veracruz and Monterrey), health institution (IMSS: Mexican Social Security Institute [Reference category]; ISSSTE: Institute of Security and Social Services of State Workers; SS Ministry of Health), daily total consumption of calories (Kcal). Model 1 is stratified by waist tertiles. Model 2 is stratified by hip tertiles. Model 3 is stratified by WHR tertiles. Model 4 is stratified by groups of silhouette trajectory. Hormone concentrations and calorie consumption were standardized to allow interpreting the odds of breast cancer development per increment of standard deviation Z=(x-μ)/σ

^3^ Logistic model in postmenopausal women: dependent variable: breast cancer (yes/no); independent variables: TT3 (nmol/L) and TT4 (nmol/L; potential confounders: age(years), city of residence (Mexico City [reference category] Veracruz and Monterrey), health institution (IMSS: Mexican Social Security Institute [Reference category]; ISSSTE: Institute of Security and Social Services of State Workers; SS Ministry of Health),TSH (continuous), parity (Continuous), consume on average one or more alcoholic drinks a month for a year (yes/no), smoked at least 100 cigarettes in her lifetime (yes/no) and indigenous ancestry (continuous). Model 1 is stratified by waist tertile. Model 2 is stratified by hip tertile. Model 3 is stratified by WHR. Model 4 is stratified by groups of silhouette trajectory. Hormone concentrations and calorie consumption were standardized to allow interpreting the odds of breast cancer development per increment of standard deviation Z=(x-μ)/σ

^4^ TT3 (Mean 1.7 SD 0.5); TT4 (Mean 103.4 SD 27.3)

Table S2. Association between thyroid function tests and BC modified by anthropometric variables. CAMA Study^1^. Mexico 2004-2007

|  |  |  | **Premenopausal women^2^** | | |  | **Postmenopausal women^3^** | | |
| --- | --- | --- | --- | --- | --- | --- | --- | --- | --- |
|  |  |  | **Case/control** | **OR** | **95% IC** |  | **Case/control** | **OR** | **95% IC** |
| **Multiple model 1 stratified by waist tertiles** | | |  |  |  |  |  |  |  |
|  | Tertil 1 (<93.05 cm) | | 61/46 |  |  |  | 119/153 |  |  |
|  |  | TT3^4^ |  | 0.02 | 0.00 - 0.11 |  |  | 0.19 | 0.12 - 0.30 |
|  |  | TT4^4^ |  | 4.10 | 1.32 - 12.76 |  |  | 3.10 | 1.94 - 4.94 |
|  | Tertil 2 (93.06-103.05 cm) | | 40/51 |  |  |  | 145/158 |  |  |
|  |  | TT3^4^ |  | 0.03 | 0.01 - 0.16 |  |  | 0.17 | 0.11 - 0.28 |
|  |  | TT4^4^ |  | 15.45 | 3.56 - 67.13 |  |  | 2.43 | 1.55 - 3.83 |
|  | Tertil 3 (>103.06 cm) | | 28/42 |  |  |  | 111/181 |  |  |
|  |  | TT3^4^ |  | 0.02 | 0.00 - 0.12 |  |  | 0.11 | 0.06 - 0.19 |
|  |  | TT4^4^ |  | 6.85 | 1.69 - 27.82 |  |  | 3.64 | 2.18 - 6.09 |
|  | p value for interaction between TT3 and waist tertiles | |  | 0.04 |  |  |  | 0.74 |  |
|  | p value for interaction between TT4 and waist tertiles | |  | 0.89 |  |  |  | 0.51 |  |
| **Multiple model 2 stratified by hip tertiles** | | |  |  |  |  |  |  |  |
|  | Tertil 1 (<103.00 cm) | | 66/44 |  |  |  | 126/162 |  |  |
|  |  | TT3^4^ |  | 0.02 | 0.003 - 0.10 |  |  | 0.14 | 0.09 - 0.23 |
|  |  | TT4^4^ |  | 9.92 | 2.78 - 35.44 |  |  | 2.17 | 1.34 - 3.50 |
|  | Tertil 2 (103.01-112.00cm) | | 32/51 |  |  |  | 132/161 |  |  |
|  |  | TT3^4^ |  | 0.05 | 0.01 - 0.19 |  |  | 0.17 | 0.10 - 0.28 |
|  |  | TT4^4^ |  | 8.18 | 2.46 - 27.22 |  |  | 3.58 | 2.16 - 5.95 |
|  | Tertil 3 (≥112.03 cm) | | 30/46 |  |  |  | 118/171 |  |  |
|  |  | TT3^4^ |  | 0.001 | 0.00005 - 0.04 |  |  | 0.15 | 0.09 - 0.24 |
|  |  | TT4^4^ |  | 3.2 | 0.40 - 25.40 |  |  | 3.47 | 2.12 - 5.68 |
|  | p value for interaction between TT3 and hip tertiles | |  | 0.17 |  |  |  | 0.49 |  |
|  | p value for interaction between TT4 and hip tertiles | |  | 0.29 |  |  |  | 0.02 |  |

^1^ CAMA: Risk factors for breast cancer in Mexico: mammographic patterns, peptide C, and growth factors, a multicenter study

^2^ Logistic model in premenopausal women: dependent variable: breast cancer (yes/no); independent variables: TT3 (nmol/L) and TT4 (nmol/L); potential confounders: All models are adjusted by: age (years), city of residence (Mexico City [reference category] Veracruz and Monterrey), health institution (IMSS: Mexican Social Security Institute [Reference category]; ISSSTE: Institute of Security and Social Services of State Workers; SS Ministry of Health), daily total consumption of calories (Kcal). Model 1 is stratified by waist tertiles. Model 2 is stratified by hip tertiles. Model 3 is stratified by WHR tertiles. Model 4 is stratified by groups of silhouette trajectory. Hormone concentrations and calorie consumption were standardized to allow interpreting the odds of breast cancer development per increment of standard deviation Z=(x-μ)/σ

^3^ Logistic model in postmenopausal women: dependent variable: breast cancer (yes/no); independent variables: TT3 (nmol/L) and TT4 (nmol/L; potential confounders: age(years), city of residence (Mexico City [reference category] Veracruz and Monterrey), health institution (IMSS: Mexican Social Security Institute [Reference category]; ISSSTE: Institute of Security and Social Services of State Workers; SS Ministry of Health),TSH (continuous), parity (Continuous), consume on average one or more alcoholic drinks a month for a year (yes/no), smoked at least 100 cigarettes in her lifetime (yes/no) and indigenous ancestry (continuous). Model 1 is stratified by waist tertile. Model 2 is stratified by hip tertile. Model 3 is stratified by WHR. Model 4 is stratified by groups of silhouette trajectory. Hormone concentrations and calorie consumption were standardized to allow interpreting the odds of breast cancer development per increment of standard deviation Z=(x-μ)/σ

^4^ TT3 (Mean 1.7 SD 0.5); TT4 (Mean 103.4 SD 27.3)

Table S2. Association between thyroid function tests and BC modified by anthropometric variables. CAMA Study^1^. Mexico 2004-2007 (*Cont)*

|  |  |  | **Premenopausal women^2^** | | |  | **Postmenopausal women^3^** | | |
| --- | --- | --- | --- | --- | --- | --- | --- | --- | --- |
|  |  |  | **Case/control** | **OR** | **95% IC** |  | **Case/control** | **OR** | **95% IC** |
| **Multiple model 3 stratified by WHR tertiles** | | |  |  |  |  |  |  |  |
|  | Tertil 1 (WHR <.888) | | 48/49 |  |  |  | 135/148 |  |  |
|  |  | TT3^4^ |  | 0.0008 | 0.00002 - 0.03 |  |  | 0.17 | 0.11 - 0.28 |
|  |  | TT4^4^ |  | 9.12 | 1.55 - 53.70 |  |  | 2.97 | 1.84 - 4.80 |
|  | Tertil 2 (WHR 0.888-.936) | | 47/47 |  |  |  | 106/158 |  |  |
|  |  | TT3^4^ |  | 0.05 | 0.01 - 0.17 |  |  | 0.15 | 0.09 - 0.25 |
|  |  | TT4^4^ |  | 6.61 | 2.05 - 21.31 |  |  | 4.43 | 2.38 - 8.27 |
|  | Tertil 3 (WHR >.936) | | 33/43 |  |  |  | 135/185 |  |  |
|  |  | TT3^4^ |  | 0.02 | 0.00 - 0.15 |  |  | 0.13 | 0.08 - 0.22 |
|  |  | TT4^4^ |  | 9.78 | 2.45 - 39.04 |  |  | 2.46 | 1.60 - 3.80 |
|  | p value for interaction between TT3 and WHR tertiles | | | 0.04 |  |  |  | 0.38 |  |
|  | p value for interaction between TT4 and WHR tertiles | | | 0.33 |  |  |  | 0.02 |  |
| **Multiple model 4 stratified by silhouette trajectory** | | |  |  |  |  |  |  |  |
|  | Group 1. Constantly low | | 16/26 |  |  |  | 58/88 |  |  |
|  |  | TT3^4^ |  | 0.003 | 0.00004 - 0.38 |  |  | 0.15 | 0.07 - 0.31 |
|  |  | TT4^4^ |  | 72.82 | 1.53 - 3,461.71 |  |  | 3.87 | 1.72 - 8.73 |
|  | Group 2. Constantly mid-range | | 50/52 |  |  |  | 153/185 |  |  |
|  |  | TT3^4^ |  | 0.03 | 0.004 - 0.13 |  |  | 0.15 | 0.09 - 0.23 |
|  |  | TT4^4^ |  | 4.14 | 1.35 - 12.66 |  |  | 2.12 | 1.44 - 3.13 |
|  | Group 3. Moderate increase | | 25/28 |  |  |  | 80/112 |  |  |
|  |  | TT3^4^ |  | 0.01 | 0.0002 - 0.18 |  |  | 0.13 | 0.07 - 0.25 |
|  |  | TT4^4^ |  | 32.52 | 2.93 - 360.56 |  |  | 2.53 | 1.37 - 4.66 |
|  | Group 4. Strong Increase | | 36/34 |  |  |  | 85/104 |  |  |
|  |  | TT3^4^ |  | 0.01 | 0.00 - 0.11 |  |  | 0.18 | 0.10 - 0.33 |
|  |  | TT4^4^ |  | 2.37 | 0.53 - 10.64 |  |  | 4.57 | 2.36 - 8.86 |
|  | Group 5. Constantly High | | 3/6 |  |  |  | 15/13 |  |  |
|  |  | TT3^4^ |  | - | - |  |  | - | - |
|  |  | TT4^4^ |  | - | - |  |  | - | - |
|  | p value for interaction between TT3 and silhouette trajectory | | | 0.58 |  |  |  | 0.36 |  |
|  | p value for interaction between TT4 and silhouette trajectory | | | 0.58 |  |  |  | 0.02 |  |

^1^ CAMA: Risk factors for breast cancer in Mexico: mammographic patterns, peptide C, and growth factors, a multicenter study

^2^ Logistic model in premenopausal women: dependent variables: breast cancer (yes/no); independent variables: TT3 (nmol/L) and TT4 (nmol/L); potential confounders: all models are adjusted by: age (years), city of residence (Mexico City [reference category], Veracruz and Monterrey), health institution (IMSS: Mexican Social Security Institute [Reference category]; ISSSTE: Institute of Security and Social Services of State Workers; SS: Ministry of Health), daily total consumption of calories (Kcal). Model 1 is stratified by waist tertiles. Model 2 is stratified by hip tertiles. Model 3 is stratified by WHR tertiles. Model 4 is stratified by groups of silhouette trajectory. Hormone concentrations and calorie consumption were standardized to allow interpreting the odds of breast cancer development per increment of standard deviation Z=(x-μ)/σ

^3^ Logistic model in postmenopausal women: dependent variable: breast cancer (yes/no); Independent variables: TT3 (nmol/L) and TT4 (nmol/L; potential confounders: All models are adjusted by age(years), city of residence (Mexico City [reference category], Veracruz and Monterrey), health institution health institution (IMSS: Mexican Social Security Institute [Reference category]; ISSSTE: Institute of Security and Social Services of State Workers; SS: Ministry of Health), TSH (continuous), parity (Continuous) , consumed on average one or more alcoholic drinks a month for a year (yes/no), smoked at least 100 cigarettes in her lifetime (yes/no) and indigenous ancestry (continuous). Model 1 is stratified by waist tertiles. Model 2 is stratified by hip tertiles. Model 3 is stratified by WHR tertiles. Model 4 is stratified by groups of silhouette trajectory. Hormone concentrations and calorie consumption were standardized to allow interpreting the odds of breast cancer development per increment of standard deviation Z=(x-μ)/σ

^4^ TT3 (Mean 1.7 SD 0.5); TT4 (Mean 103.4 SD 27.3)
